# The impact of submucosal PRP injection on wound healing after endoscopic sinus surgery: a randomized clinical trial

**DOI:** 10.1007/s00405-024-08483-6

**Published:** 2024-02-09

**Authors:** Konstantina Dinaki, Nikolaos Grigoriadis, Ioannis S. Vizirianakis, Jannis Constantinidis, Stefanos Triaridis, Petros Karkos

**Affiliations:** 1 1st Academic ORL Department, AHEPA Hospital, Aristotle University of Thessaloniki, Thessaloniki, Greece; 2 Laboratory of Pharmacology, School of Pharmacy, Aristotle University of Thessaloniki, Thessaloniki, Greece; 3 Department of Health Sciences, School of Life and Health Sciences, University of Nicosia, Nicosia, Cyprus

**Keywords:** Platelet-rich plasma, Endoscopic sinus surgery, Chronic rhinosinusitis

## Abstract

**Purpose:**

Chronic rhinosinusitis (CRS) is a prevalent chronic disease observed on a global scale. The utilization of endoscopic sinus surgery (ESS) has gained significant recognition as an effective intervention for individuals with CRS and nasal polyps who have not responded to conventional treatments. The need (or not) for revision surgery frequently relies on the promotion of optimal wound healing. The impact of platelet-rich plasma (PRP) on tissue healing has been extensively examined in various surgical fields.

**Methods:**

The present prospective study involved 30 patients suffering with nasal polyposis who underwent endoscopic sinus surgery. 15 patients were assigned to the PRP group, and 15 patients to the control group. The clinical follow-up of the patients took place at specific intervals, at weeks 1, 2, 3, 4, 8, and 12 after the surgical procedure. The evaluator identified the existence of adhesions, crusting, bleeding, granulation and infection using a visual analogue scale score. The patients also completed the SNOT 22 questionnaire prior to surgery and at each postoperative visit.

**Results:**

The present study observed a lower incidence of adhesion, infection, hemorrhage and granulation in the PRP group. Furthermore, a statistically significant difference was detected between the groups.

**Conclusion:**

Based on the findings of the present investigation, it seems that platelet-rich plasma (PRP) is beneficial on wound healing during the early stages following the surgical procedure. The technique is characterized by its limited invasiveness, which contributes to its low risk profile and the achievement of clinically good outcomes.

## Introduction

Chronic rhinosinusitis (CRS) is one of the most prevalent chronic disorders, with an estimated prevalence of between 5 to 12% in western countries [1] and 10–15% globally [2, 3]. Endoscopic sinus surgery (ESS) is a procedure that has gained widespread acceptance as a treatment option for medically resistant chronic rhinosinusitis and nasal polyps [4]. The primary goals of ESS are to “open” the sinuses, remove any lesions and restore ventilation and drainage so as to reduce the recurrence of sinusitis and improve patients’ symptoms. In ESS, one of the most critical challenges is problematic healing that could potentially result in recurrence of the disease and a need for revision surgery. The recovery process following ESS is quite unpredictable and difficult to anticipate. Various techniques can be employed to enhance this, such as implementing meticulous postoperative care. It is important to note that bleeding and crusting might potentially impact or facilitate the formation of synechiae and granulation. However, it is not proven that they play a part in the recurrence of the polyposis. The rate of CRS recurrence after ESS varies in the literature, but wound healing has been regarded as a crucial factor in determining the procedure's efficacy [5]. Complications of healing such as bleeding, inflammation and adhesion in the middle meatus are the most common ones that might occur after an ESS, emphasizing the necessity for innovations in postoperative care [6, 7].

Platelet-rich plasma (PRP) is a component of plasma developed by dual-speed centrifugation of whole blood. It has a concentration of platelets five times greater than normal blood, at least 1,000,000 platelets/μl in 5 ml of plasma [8]. Platelets are best known for their function in hemostasis, but they also promote and control wound healing via a variety of growth factors, cytokines, and bioactive molecules [9, 10]. During healing, growth factors have major impacts on cell regulation, differentiation, proliferation, migration, chemotaxis, angiogenesis, matrix formation and collagen synthesis [10, 11]. PRP acts by degranulating the alpha granules of platelets, secreting a number of growth factors that promote early wound healing. Alpha granules comprise a variety of growth factors, including platelet-derived growth factor (PDGF), vascular endothelial growth factor (VEGF), fibroblast growth factor (FGF), epidermal growth factor (EGF), transforming growth factor (TGF) and insulin like growth factor (IGF) [12]. Numerous clinical studies have examined the application of platelet concentrations, such as PRP and PRF (platelet-rich fibrin) in the field of ENT but only a few of them investigated their use in endoscopic sinus surgery [3, 10, 12–15]. In this study, we aimed to evaluate the effect of submucosal application of PRP after ESS. We hypothesized that PRP would have a beneficial effect on wound healing in the early postoperative period, resulting in a quicker amelioration of patients' symptoms and a decreased need for revision surgery.

## Patients and methods

### Patients

This study was designed as a prospective randomized controlled clinical trial. Prior to the beginning of the study, approval was obtained from the local ethics committee. All patients provided their informed consent.

**Fig. 1:**
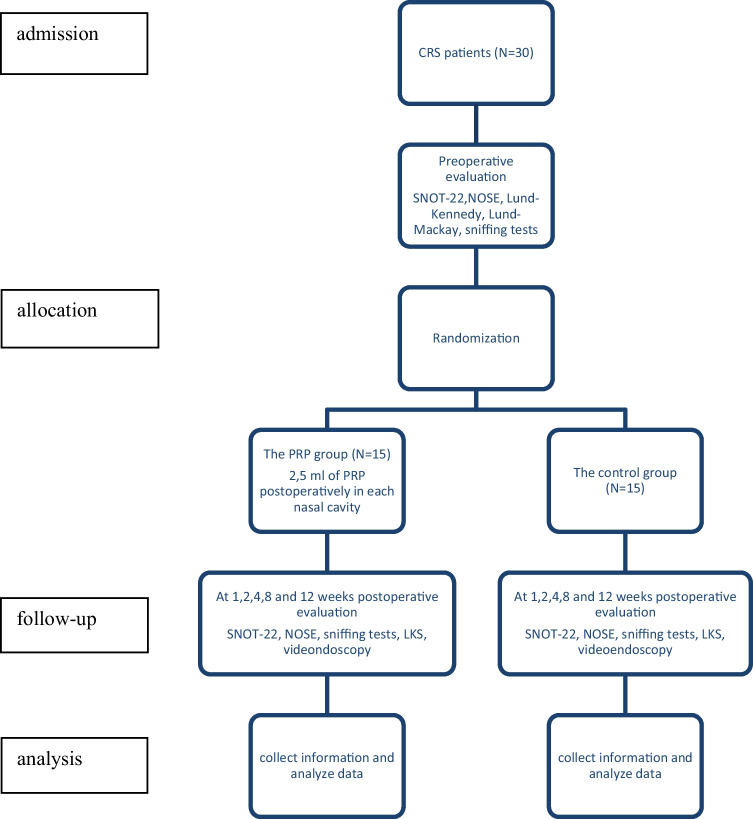
CONSORT diagram of the randomized controlled trial. *CONSORT* Consolidated Standards of Reporting Trials, *CRS* chronic rhinosinusitis, *SNOT-22* Sino-Nasal Outcome Test 22, *LKS* Lund–Kennedy Scoring system

The study was conducted between 2019 and 2023. Eligible participants were adults undergoing bilateral ESS for CRS refractory to medical management. The inclusion criteria were: age > 18, persistent bilateral CRS, a difference < 2 points in the preoperative Lund–Mackay score between sides. Exclusion criteria included a preoperative Lund–Mackay score difference of more than two points between sides, unilateral polyps, a history of previous ESS or nasal polyp surgery and excision of the middle turbinates, a history of underlying immunologic diseases and patients with a known risk for bleeding. Preoperative CT scans were evaluated using the Lund–Mackay scoring system. Thirty patients were enlisted and surgically treated. 15 patients were assigned to the PRP group, and 15 patients were assigned to the control group. The trial protocol is summarized in the consolidated standards of reporting trials (CONSORT) flow diagram (Fig. 1).

### PRP preparation

**Fig. 2:**
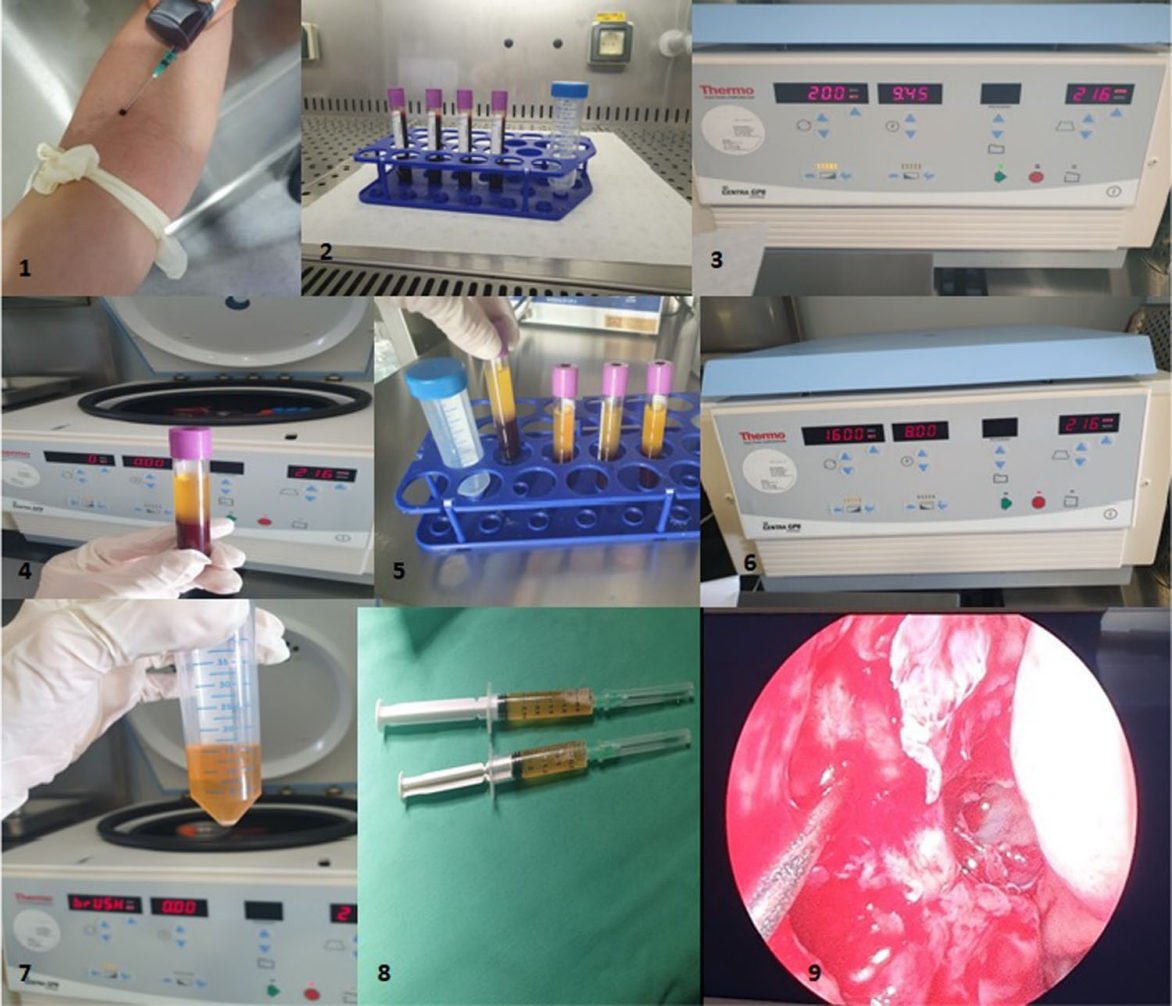
1–2: peripheral intravenous blood collection (40 ml), 3: first centrifugation in 200 speed/relative centrifugal force (RCF) for 12 min (soft spin), 4–5: upper layer and buffy coat collection with a Pasteur pipette, 6: second centrifugation at 1600 speed/ RCF for 8 min (hard spin), 7: removal of the subsequent platelet-poor plasma, 8: a total of 5 ml of PRP is obtained, 9: 2.5 ml of PRP is injected submucosally in the ethmoidectomy cavity in the middle meatus and around the antrostomy

PRP is produced by dual-speed centrifugation. Blood is separated into red blood cells, a buffy coat and platelet-poor plasma during the first centrifugation (soft spin). The second centrifugation process (hard spin) separates further the plasma into layers of platelet-rich plasma and platelet-poor plasma [12]. The PRP was prepared in the Biogenea Pharmaceuticals Laboratory. Prior to the beginning of the surgery, 40 ml of blood was obtained intravenously and collected in sterile vacuum tubes coated with anticoagulant. First, the blood was centrifuged at 200 speed/relative centrifugal force (RCF) for 12 min (soft spin) to separate the red blood cells from the plasma and platelets. After centrifugation, three layers were obtained: plasma on top, buffy coat with platelets and white blood cells in the middle, and red blood cells at the bottom. The upper layer and the buffy coat were collected with a Pasteur pipette and centrifuged at 1600 speed/RCF for 8 min (hard spin). Following the removal of the subsequent platelet-poor plasma, there was a total of 5 ml of PRP left, which was transferred into a sterile tube. (Fig. 2) Using an automated hematology analyzer, platelets in PRP samples were accurately counted to ensure adequate platelet suspension (> 10^6^ platelets/μl).

### Surgical procedure and postoperative care

All patients underwent bilateral ESS with power instrumentation, mucosal sparing, and bilateral middle turbinate preservation at the University General Hospital of Thessaloniki, AHEPA, by the same surgeon under general anesthesia. At the end of the surgery and after hemostasis was achieved, 2.5 ml of PRP were injected submucosally in the ethmoidectomy cavity in the middle meatus and around the antrostomy in each nasal cavity of the patients in the PRP group. All patients were packed with a small nasal tampon of 4 cm length in each nasal cavity.

All patients were discharged on the first postoperative day and all were prescribed prophylactic antibiotics (amoxicillin clavulanic acid 875 + 125 mg twice per day for 10 days). Packing was removed on the first postoperative day and patients were instructed to commence nasal irrigation with normal saline and mometasone nasal spray.

### Outcome measures

All patients were scheduled for follow-up visits at postoperative weeks 1, 2, 4, 8 and 12. All patients underwent video endoscopy to evaluate adhesions, hemorrhage, crusting, granulation and infection parameters. The nasal endoscopy was performed by a physician other than the operating surgeon, and all parameters were graded using an ordinal scale modified from the existing grading scales. (Table 1) [16–18]. Patients completed the SNOT-22 questionnaire prior to surgery and at each postoperative visit. On the SNOT-22 questionnaire, patients evaluate 22 different symptoms related to nasal function, physical state, and emotional characteristics on a scale ranging from 0 to 5 with 0 indicating no symptoms and 5 indicating the most severe symptoms [19, 20]. The sense of smell was checked using scent markers, the Greek version of sniffing sticks, at 4, 8 and 12 week postoperatively [21]. The identification of odors is evaluated using 16 common odors. For each odor, the test subject must select the correct descriptor from a list of four. The range of identification scores is between 0 and 16.

**Table 1:** Grading scale for adhesion, bleeding, crusting, infection and granulation

Grading scale	Description
Adhesion	
0	No adhesion
1	Mild (easy to detach)
2	Moderate (hard to detach)
3	Severe (need synechiolysis)
Bleeding	
0	No bleeding
1	Minimal (confined to nasal cavity)
2	Moderate (out of nasal cavity)
3	Severe (need repacking or cauterization)
Crusting	
0	Absent
1	Mild (without obliteration of the ethmoid cavity)
2	Moderate (partial obliteration of the ethmoid cavity)
3	Severe (total obliteration of the ethmoid cavity)
Infection	
0	No visible evidence of infection
1	Mild mucopurulent discharge
2	Moderate mucopurulent discharge
3	Gross mucopurulent discharge with obvious frank infection
Granulation	
0	Absent
1	Mild
2	Moderate
3	Severe

### Statistical analysis

Continuous variables that followed a normal distribution are presented as mean ± standard deviation, while continuous variables that did not follow a normal distribution are presented as medians and 25th and 75th percentiles. All categorical variables are presented as frequencies and percentages (%). Comparison of means from two normally distributed continuous variables was performed using the Student’s *t*-test, and comparison of medians from two non-normally distributed continuous variables was performed using the Mann–Whitney *U* test. Statistical analysis was performed with SPSS version 25.0 (IBM, Armonk, NY, USA). Statistical significance was set at *p* < 0.05.

## Results

A total of 30 people participated in the survey. All of them underwent ESS with nasal polypectomy. In 15 patients (intervention group) with a mean age of 45.47 ± 13.24, PRP was placed after the operation and in the remaining 15 (control group) with a mean age of 52.20 ± 7.64 nothing was placed. Each patient underwent surgery on both sides of the nose. The intervention group was given 2.5 ml of PRP on each side right and left (30 nasal cavities) and the control group was given nothing on either side (30 nasal cavities).

**Fig. 3:**
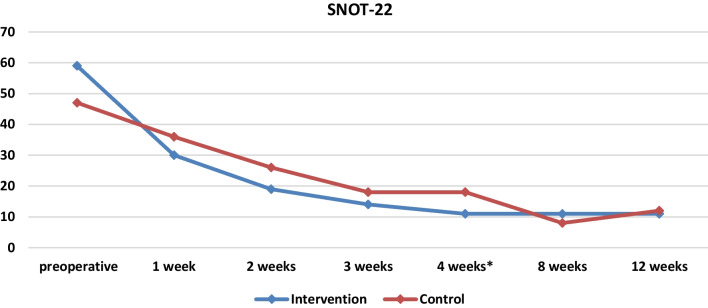
Medians and comparisons for intervention group and control group of SNOT-22 score at each time point

**Fig. 4:**
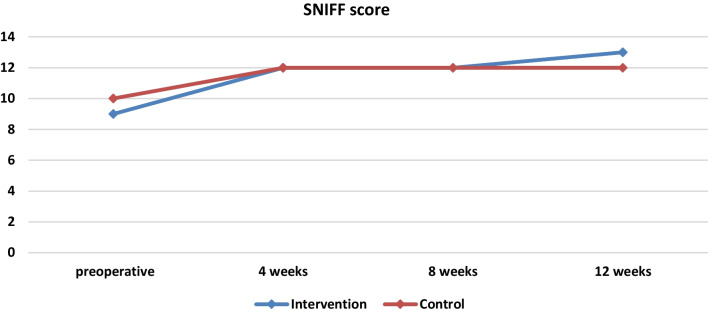
Medians and comparisons for intervention group and control group of SNIFF score at each time point

**Fig. 5:**
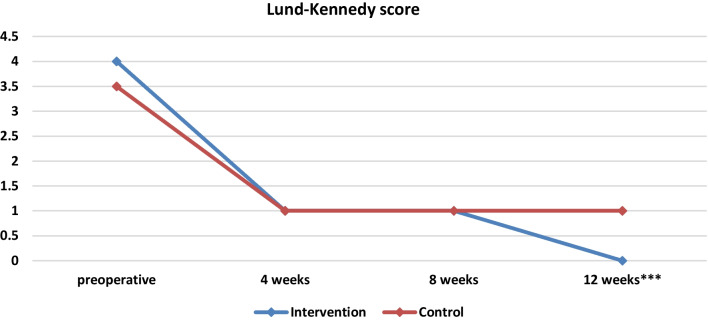
Medians and comparisons for the intervention group and the control group of Lund–Kennedy score (both sides) at each time point

By first studying the parameters preoperatively, the median scores of the SNOT-22 scale preoperatively for the intervention group amounted to 9 (6 14 the 25th and 75th percentile accordingly) and for the control group to 10 (6 12) without being statistically significantly different (*p* = 0.512). Accordingly, no differences are observed in the medians for the intervention and control groups for both the SNIFF variable and the Lund–Kennedy scale (Table 2, Figs. 3, 4, 5).

**Table 2:** Baseline demographics and clinical information

	Intervention (*Ν* = 15)	Control (*Ν* = 15)	*p*-values
Age (years)	45.47 ± 13.24	52.20 ± 7.64	*p* = 0.102
SNOT-22 score	59 (39 75)	47 (30 73)	*p* = 0.512
SNIFF	9 (6 14)	10 (6 12)	*p* = 0.870
Lund–Kennedy score (right side)	4 (3 5)	3 (2 4)	*p* = 0.233
Lund–Kennedy score (left side)	4 (3 5)	4 (3 4)	*p* = 0.539
Lund–Kennedy score (both sides)	4 (3, 5)	4 (3, 5)	*p* = 0.168

Table 3 shows the scores for all variables preoperatively and at follow-up for all sides. There are statistical significant differences in SNOT-22 score after 4 weeks of the operation, in VAS score and crusting specifically in every time point, in 1st and 2nd week for bleeding, in 8th and 12th week for granulation and in 12th week for Lund–Kennedy score.

**Table 3:** Median, 25th and 75th percentiles and Mann–Whitney *U* comparisons for the intervention group and the control group for all variables at each respective time point (both sides-30 nasal cavities)

	Preoperative	Follow-up (weeks)					
		1	2	3	4	8	12
SNOT-22 score							
Intervention	59 (39 75)	30 (14 44)	19 (11 37)	14 (10 19)	11 (5 13)	11 (6 15)	11 (5 15)
Control	47 (30 73)	36 (12 48)	26 (13 37)	18 (10 36)	18 (9 28)	8 (6 22)	12 (7 22)
U	96.0	100.0	89.5	78.5	63.0	105.0	93.0
P	0.512	0.624	0.345	0.161	0.041*	0.775	0.436
SNIFF score							
Intervention	9 (6 14)				12 (9 15)	12 (9 15)	13 (10 14)
Control	10 (6 12)				12 (9 13)	12 (10 14)	12 (9 14)
U	108.0				95.5	102.5	97.5
P	0.87				0.486	0.683	0.539
VAS score							
Intervention		2 (1, 2)	3 (2, 4)	2 (1, 4)	1 (0, 2)	0 (0, 1)	0 (0, 1)
Control		3 (3, 5)	5 (3, 6)	3.5 (3, 4)	3 (2, 3)	2 (1, 2)	2 (1, 2)
U		177.5	231.5	253.0	183.0	225.5	238.0
P		0.000037***	0.001**	0.003**	0.000045***	0.001**	0.001**
Adhesion							
Intervention		0 (0, 0)	0 (0, 1)	0 (0, 1)	0 (0, 1)	0 (0, 1)	0 (0, 1)
Control		0 (0, 1)	0 (0, 1)	0 (0, 1)	0 (0, 1)	0 (0, 1)	0 (0, 1)
U		360.0	436.0	378.0	381.0	390.0	420.0
P		0.069	0.809	0.197	0.226	0.288	0.587
Bleeding							
Intervention		0 (0, 1)	0 (0, 0)	0 (0, 0)	0 (0, 0)	0 (0, 0)	0 (0, 0)
Control		1 (0, 1)	1 (0, 1)	0 (0, 0)	0 (0, 0)	0 (0, 0)	0 (0, 0)
U		280.0	253.5	420.0	405.0	450.0	450.0
P		0.004**	0.000328***	0.393	0.078	1.000	1.000
Crusting							
Intervention		1 (1, 2)	1 (1, 1)	1 (1, 1)	0 (0, 1)	0 (0, 0)	0 (0, 0)
Control		2 (2, 2)	2 (1, 2)	2 (1, 2)	1 (1, 1)	0 (0, 1)	0 (0, 1)
U		180.5	225.0	241.0	160.0	310.0	345.0
P		0.000006***	0.000173***	0.001**	0.000002***	0.011*	0.021*
Infection							
Intervention		0 (0, 0)	0 (0, 1)	0 (0, 1)	0 (0, 0)	0 (0, 0)	0 (0, 0)
Control		0 (0, 1)	1 (0, 1)	0 (0, 1)	0 (0, 0)	0 (0, 0)	0 (0, 0)
U		375.0	360.0	420.0	450.0	450.0	450.0
P		0.120	0.121	0.576	1.000	1.000	1.000
Granulation							
Intervention		0 (0, 0)	1 (1, 2)	1 (1, 1)	0 (0, 1)	0 (0, 1)	0 (0, 1)
Control		0 (0, 0)	1.5 (0, 2)	1 (1, 2)	1 (0, 1)	1 (0, 1)	1 (0, 1)
U		450.0	388.0	426.0	360.0	315.0	284.0
P		1.000	0.327	0.695	0.121	0.021*	0.005**
Lund–Kennedy score							
Intervention	0 (0, 0)				1 (0, 1)	1 (0, 1)	0 (0, 0)
Control	0 (0, 0)				1 (0, 1)	1 (0, 1)	1 (0, 1)
U	450.0				424.0	426.5	229.5
P	1.000				0.669	0.694	0.000215***

Statistical significance was set at *p* < 0.05 and the results that show statistical significant difference are in bold

^*^p < 0.05, **p < 0.01, ***p < 0.001

In the first week statistically significant differences were found between the two groups in terms of bleeding and crusting. Crusting was less prominent in PRP group. Eighty percent, 80% (*n* = 24) of the sides of the control group had moderate crusting, while 36.6% (*n* = 11) had moderate crusting on the PRP group. There was a statistically significant difference in the two groups as shown in Table 3 (*p* = 0.000006). In the control group 66.6% (*n* = 20) of the nasal cavities presented minimal bleeding, while 33.3% of the sides in the PRP group presented minimal bleeding as well (*p* = 0.004). In the second week the same results were observed as statistically significant differences were found between the two groups in terms of crusting (*p* = 0.000173) and bleeding (*p* = 0.000328). In the control group fifty percent (*n* = 15) of nasal cavities presented minimal bleeding and 56.6% (*n* = 17) had moderate crusting, while 10% of the sides in the PRP group (*n* = 3) presented minimal bleeding and 16.6% (*n* = 5) had moderate crusting.

**Fig. 6:**
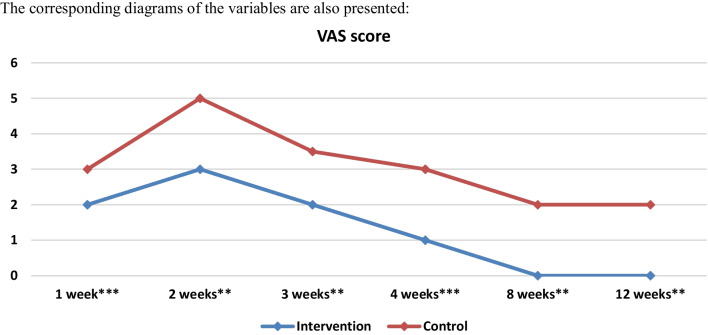
Medians and comparisons for the intervention group and the control group of the VAS score (both sides) at each time point

In the third and fourth weeks statistically significant differences were observed only in crusting (*p* = 0.001 in the third week and *p* = 0.000002 in the fourth week). In terms of adhesions, infection and granulation no statistically important differences were observed during the first four weeks between the two groups (*p* > 0.05). However, the total VAS score was statistically significantly lower in the PRP group in every postoperative week as shown in Tables 3 and Fig. 6.

In the eighth week statistically important differences were observed between the two groups in terms of crusting and granulation. In the control group 6.6% (*n* = 2) of the nasal cavities presented moderate crusting and 40% (*n* = 40) presented mild crusting, while in the PRP group only 16.6% (*n* = 5) presented mild crusting. Additionally in the PRP group only 30% (*n* = 9) had mild granulation while in the control group 60% (*n* = 18) of the sides presented mild granulation. In week 12 same results were observed with statistically significant differences between the two groups in terms of crusting and granulation (*p* = 0.021 and *p* = 0.005). In the control group 86.6% (*n* = 26) presented no or mild granulation while in PRP group 100% (*n* = 30) had no or mild granulation. In the 8 and 12 weeks in terms of bleeding, adhesions and infection no statistically important differences were observed between the two groups. However, as in the first 4 weeks, the total VAS score was significantly different between the two groups in week 8 and 12 (Table 3, Fig. 6).

In week 4, statistically significant difference was reported between the two groups in SNOT 22 score and in week 12 statistically significant difference was observed in Lund–Kennedy score. As far as olfactory function is concerned, the improvement in sniff scores in both groups showed no statistically significant difference during the whole postoperative period (*p* > 0.05).

## Discussion

PRP has proven to be efficient on wound healing across various fields of medicine and surgery. ESS remains the gold standard as a treatment option for CRS resistant to medical treatment [4]. The primary objective of ESS is restoration of nasal ventilation, preservation of essential nasal structures (particularly the middle turbinate) and rectification of abnormal nasal anatomy. In order to achieve these goals, it is essential to conserve as much sinus mucosa as possible throughout the operation [22, 23]. In ESS, one of the greatest challenges is preventing healing complications that could lead to recurrence of the disease necessitating revision surgery. Wound healing process has been considered a crucial factor in determining the effectiveness of the procedure. Healing issues such as bleeding, inflammation and adhesions in the middle meatus are some of the most prevalent ones that could develop after an ESS, highlighting the need for meticulous postoperative care [6, 7].

In the nasal mucosa, wound healing is a well-organized process that involves inflammation, cell proliferation, matrix deposition, and remodeling [10, 24]. This process is controlled by a wide variety of growth factors and cytokines. The process consists of four phases: the phase of coagulation (5–10 min), the phase of inflammation (24–48 h), the phase of tissue formation (4 days) and the phase of tissue remodeling (6 months) [10, 24]. Observing the mucosal healing following sinus surgery using videoscopy, four overlapping phases of wound healing that occur after sinus surgery are distinguished [24]. The initial stage, which lasts for seven to twelve days after the injury, is characterized by blood crusts coating the whole wound. The second phase, which consists of the creation of granulation tissue, can last from two to four weeks. A third edematous phase and then a phase of macroscopic normalization (lasting from the 12th to the 18th week) bring this process to a close [24].

Platelet-rich products, such as platelet-rich plasma (PRP) and platelet-rich fibrin (PRF), have been shown to have favorable effects on wound healing because of their high concentrations of platelets, cytokines, and growth factors [12, 25]. These substances stimulate cell proliferation, promote wound healing and hemostasis and reduce scarring [26].

Despite the widespread application of PRP in the field of otolaryngology, only a few clinical studies have investigated the effectiveness of PRP in ESS [3, 13–15, 27]. While Mohebbi et al. [14] came to the conclusion that the use of PRP after ESS may be effective in reducing symptoms subjectively, Tabrizi et al. [13] observed that there was no short-term effect on the recovery of olfactory function in patients with sinonasal polyps after an intranasal injection of PRP following their surgery. The effects of PRF on wound healing following ESS were investigated by Sari et al. [3]. The PRF group was found to have fewer adhesions, infection, hemorrhage, granulation and frontal ostium stenosis, as well as superior overall results.

**Fig. 7:**
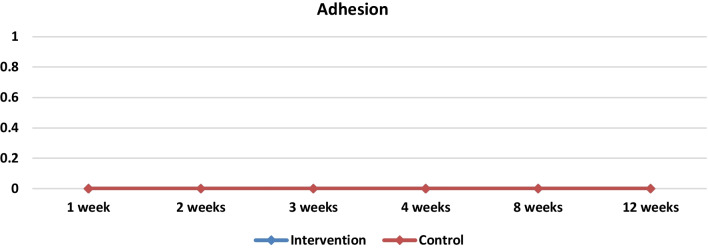
Medians and comparisons for the Adhesion intervention group and control group (both sides) at each time point

Adhesion formation is a significant complication observed in ESS. The primary factor contributing to the development of adhesions in endoscopic sinus surgery is the occurrence of non-epithelialized surfaces coming into contact and then undergoing healing as a cohesive unit. PRP promotes the process of epithelialization and serves as a preventive factor against adhesions [28]. It has been shown that PRP injection reduces intranasal adhesion and fibrosis [10]. In their animal study, Yildirim et al. discovered a substantial decrease in hydroxyproline levels within the PRP group. This observation appears to be associated with reduced collagen intensity as observed through histological examination. Consequently, these findings suggest that the healing process, specifically in relation to intranasal synechiae and fibrosis, was improved [10]. In our study, there was no statistically significant difference in adhesion between the two groups (Table 3, Fig. 7). This can be explained by the limited number of patients.

**Fig. 8:**
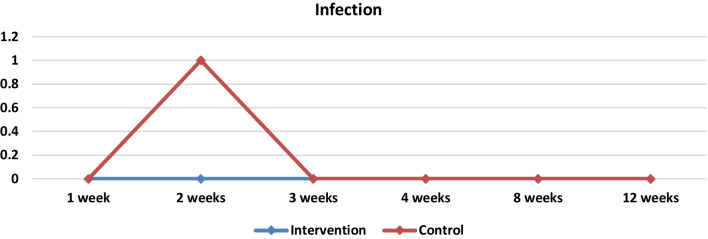
Medians and comparisons for the intervention group and the control group of infection (bothsides) at each time point

Numerous investigations have provided evidence of the antibacterial effects demonstrated by PRP [29–31]. Precise mechanisms underlying the antibacterial properties of platelet concentrates are not yet fully elucidated due to the complicated composition of these products. They produce oxygen metabolites, such as superoxide, hydrogen peroxide [32, 33] and furthermore, they possess the ability to bind, aggregate, and internalize bacteria, so they enhance the removal of pathogens from the circulatory system [34, 35]. Activated platelets have the ability to release a variety of growth factors (GFs) that are secreting platelet microbicidal proteins (PMPs) [32]. PMPs consist of many substances that exhibit antibacterial properties. They have the potential to exert their influence via several processes, which include direct interaction with the bacterial membrane, inducing alterations in membrane permeability, internalization into the cell and impeding macromolecules synthesis [35]. In our investigation, we observed that there was no statistically significant variation in infection rates between the two cohorts (Table 3, Fig. 8). This outcome may be attributed to the relatively small sample size and the absence of serious infections in either group.

**Fig. 9:**
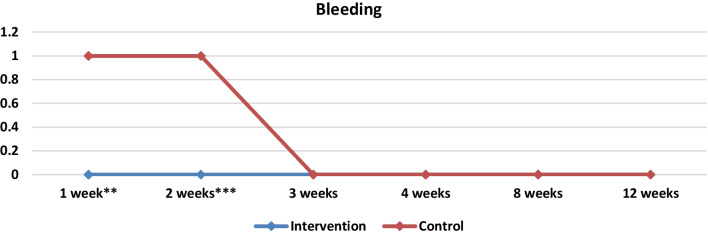
Medians and comparisons for the intervention group and the control group of bleeding (both sides) at each time point

PRP demonstrates a notable capacity for achieving hemostasis as a result of its elevated platelet content [36, 37]. In our investigation, a statistically significant difference was observed between the two groups during the initial 2-week period following the surgical procedure. The control group exhibited a higher frequency of epistaxis occurrences during the first 2 weeks (*p* = 0.004 in the first week and *p* = 0.000328 in the second week) (Table 3, Fig. 9). In the first week 66.6% (*n* = 20) of the nasal cavities studied in the control group presented minimal bleeding and this decreased to 50% (*n* = 15) in the second week, while in the PRP group 33.3% (*n* = 10) had minimal bleeding which decreased in 10% (*n* = 3) in the second week. In the long-term follow-up, neither of the two groups exhibited any instances of postoperative bleeding. The occurrence of bleeding after nasal packing removal results in patient’s discomfort. The formation of a crust following an episode of epistaxis contributes to the exacerbation of nasal obstruction symptoms and hinders the progression of the healing process.

**Fig. 10:**
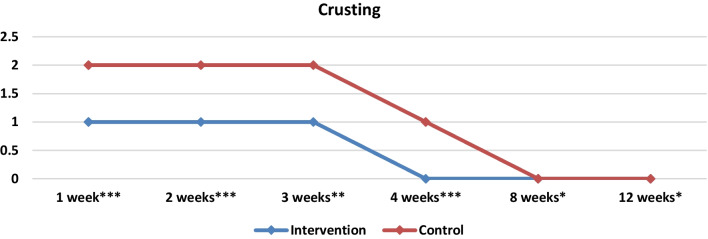
Medians and comparisons for the intervention group and the control group of crusting (both sides) at each time point

There is limited data evaluating the effect of platelet products in crusting formation after nasal surgery [15, 28]. In the few studies available in literature it seems that PRP application leads to significantly lower crusting results in nasal surgeries [15, 28]. In our study a statistically significant difference was observed between the two groups during the entire postoperative period (Table 3, Fig. 10). In the first week 80% (*n* = 24) of the sides in the control group presented moderate crusting, which decreased in 56.6% (*n* = 17) in the second week. In the PRP group 36.6% (*n* = 11) of the studied sides presented moderate crusting and this decreased to 16.6% (*n* = 5) in the second week. In the eighth week 40% (*n* = 12) of the sides in the control group presented mild crusting in contrast with 16.6% (*n* = 5) in the PRP group.

**Fig. 11:**
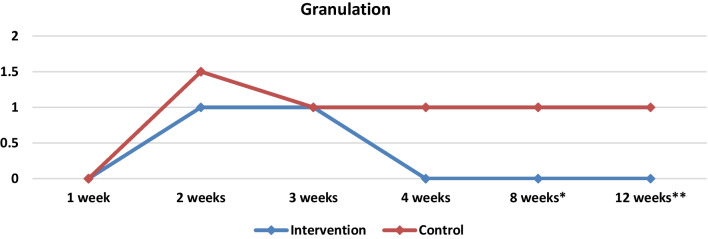
Medians and comparisons for the intervention group and the control group of granulation (bothsides) at each time point

Growth factors are essential to the process of tissue remodeling. The extension of the duration of granulation tissue signifies a lengthening of the proliferation phase in the process of wound healing [28]. Research has demonstrated that PRP has a positive impact on the healing process of chronic wounds, facilitating the formation of healthy granulation tissue and accelerating wound closure [38]. In our study less granulation was present in PRP group during the whole postoperative period and it was found to be statistically important during the 8th and 12th weeks (Table 3, Fig. 11). In week 8, 60% (*n* = 18) of the sides in the control group had mild granulation which decreased to 46.6% (*n* = 14) in week 12, while in PRP group only 30% (*n* = 9) presented mild granulation in the eighth week which decreased to 26.6% (*n* = 8) in the twelfth week.

Statistically significant differences in Visual Analog Scale (VAS) scores were observed during the whole postoperative period, indicating that the variations in each parameter examined in this study contribute to overall improved healing. (Table 3, Fig. 6).

Lund–Kennedy score was found to be significantly lower in the PRP group in the end of the 3-month follow-up, demonstrating improved outcomes in chronic rhinosinusitis in the patients in this group (Table 3, Table 12).

In recent years, a few clinical and animal studies have been conducted to examine the therapeutic potential of platelet-rich plasma in the management of anosmia [13, 39–42]. The majority of them have examined the application of PRP in the olfactory region in patients experiencing anosmia due to degeneration of the olfactory epithelium. Based on the theory that PRP possesses a substantial concentration of growth factors and neurotropic factors it is expected that PRP could serve as an efficacious neuroregenerative intervention. Consequently, the administration of PRP may potentially stimulate the regeneration of basal cells and offer a therapeutic approach for the treatment of anosmia [13]. Those studies provided highly promising outcomes in the management of anosmia [40–42]. On the other hand, there is only one study available in the literature that has investigated the use of PRP in patients suffering from anosmia caused by sinonasal polyps [13]. The PRP injection following endoscopic sinus surgery did not have any significant impact on the restoration of olfactory function. Based on the existing literature, it appears that surgical intervention continues to be the optimal approach for managing olfactory dysfunction in individuals with nasal polyps. Our study complies with the limited data currently available, since there was no statistically significant difference observed between the two groups. The improvement in sniff score observed in both groups is explained by the nasal polypectomy (Table 3, Table 11).

Our study is subject to many limitations, primarily the small sample size of the patients and the absence of double blinding. The duration of the postoperative phase was restricted to a 3-month follow-up, hence limiting the ability to assess long-term impact. Future studies with long-term follow-up and a double-blinded design would be beneficial in evaluating the impact of PRP on the healing of nasal mucosa following ESS.

## Conclusion

ESS is only a small fraction of overall management of CRS and appropriate medical treatment is necessary following surgical intervention [43]. Enhancing the clinical effectiveness involves improving the nasal mucosa's functionality, expediting wound healing and facilitating the process of mucosal epithelialization. Our study provides evidence supporting the effectiveness of PRP in promoting postoperative healing and enhancing patient satisfaction following endoscopic sinus surgery. Superior results were achieved with regard to three parameters, i.e., bleeding, crusting and granulation. However, there was no statistically significant effect on the development of adhesions and infection. We are not convinced that a favorable and prompt postoperative healing process is related to a reduced likelihood of polyp development in the future. The PRP injection is characterized by minimal invasiveness, no hazards or side effects and clinically good outcome. Additional research is necessary to validate the encouraging findings of this study.

## Data Availability

The data presented in this study are available upon request from the corresponding author.
